# The independence of intrinsic radiosensitivity as a prognostic factor for patient response to radiotherapy of carcinoma of the cervix.

**DOI:** 10.1038/bjc.1997.531

**Published:** 1997

**Authors:** C. M. West, S. E. Davidson, S. A. Roberts, R. D. Hunter

**Affiliations:** Cancer Research Campaign Department of Experimental Radiation Oncology, Paterson Institute for Cancer Research.

## Abstract

A study was made of the prognostic value of pretreatment measurements of tumour radiosensitivity (surviving fraction at 2 Gy, SF2) in 128 patients with stage I-III carcinomas of the uterine cervix undergoing radiotherapy. The median follow-up time was 47 months. In a univariate analysis stratifying patients according to the median value, radiosensitivity was a significant prognostic factor for overall survival, local control and metastasis-free survival. The 5-year survival rate for tumours with SF2 values below the median was 81% and was significantly greater than the rate of 51% for those with SF2 values above the median. In bivariate analyses, SF2 was shown to be independent of disease stage, tumour grade, patient age, colony-forming efficiency and tumour diameter. In a multivariate analysis, radiosensitivity was the most important variable and, after allowing for this, only stage was a significant independent predictor of treatment outcome. These data indicate that, in carcinoma of the cervix treated with radiotherapy, pretreatment tumour intrinsic radiosensitivity is an important prognostic parameter and contributes to prognosis independently of other established and putative parameters.


British Journal of Cancer (1997) 76(9), 1184–1190
© 1997 Cancer Research Campaign

### The independence of intrinsic radiosensitivity as a prognostic factor for patient response to radiotherapy of carcinoma of the cervix

**CML West[1], SE Davidson[2], SA Roberts[3] and RD Hunter[2]**

[1]Cancer Research Campaign Department of Experimental Radiation Oncology, Paterson Institute for Cancer Research; [2]Department of Clinical Oncology, Christie Hospital (NHS) Trust, Wilmslow Road, Manchester M20 4BX, UK; [3]Cancer Research Campaign Department of Biomathematics and Computing, Paterson Institute for Cancer Research

**Summary** A study was made of the prognostic value of pretreatment measurements of tumour radiosensitivity (surviving fraction at 2 Gy, $SF_2$) in 128 patients with stage I–III carcinomas of the uterine cervix undergoing radiotherapy. The median follow-up time was 47 months. In a univariate analysis stratifying patients according to the median value, radiosensitivity was a significant prognostic factor for overall survival, local control and metastasis-free survival. The 5-year survival rate for tumours with $SF_2$ values below the median was 81% and was significantly greater than the rate of 51% for those with $SF_2$ values above the median. In bivariate analyses, $SF_2$ was shown to be independent of disease stage, tumour grade, patient age, colony-forming efficiency and tumour diameter. In a multivariate analysis, radiosensitivity was the most important variable and, after allowing for this, only stage was a significant independent predictor of treatment outcome. These data indicate that, in carcinoma of the cervix treated with radiotherapy, pretreatment tumour intrinsic radiosensitivity is an important prognostic parameter and contributes to prognosis independently of other established and putative parameters.

**Keywords**: predictive assay; intrinsic radiosensitivity; $SF_2$; cervix cancer; radiotherapy

There are considered to be three important radiobiological factors that determine how well a tumour responds to radiotherapy: intrinsic radiosensitivity, hypoxia and proliferation. The clinical relevance of these parameters is currently receiving considerable attention, and studies have been published suggesting the potential of all three as prognostic factors for radiotherapy (West, 1994).

In carcinoma of the cervix, our own studies have indicated that tumour radiosensitivity is an important determinant of treatment outcome (West et al, 1991, 1993). In this work, radiosensitivity is measured using a soft agar clonogenic assay as surviving fraction after 2 Gy in vitro irradiation ($SF_2$). Some support for our finding has come from work on head and neck tumours, which has shown that radiosensitivity, measured using a growth assay as the initial slope of radiation survival curves ($\alpha$), significantly influenced patient outcome when a high (above median) value was used to stratify data (Girinsky et al, 1994). Other smaller studies using a variety of assays have also suggested that radiosensitive tumours are more responsive to therapy (Hinkley and Bosanquet, 1992; Ramsay et al, 1992; Vaughan et al, 1993; Shibamoto et al, 1994). Studies that have shown no relationship between tumour radiosensitivity and treatment outcome either involved the establishment of cell lines before assay (Allalunis-Turner et al, 1992; Schwartz et al, 1992; Taghian et al, 1993) or treatment with surgery plus radiotherapy (Brock et al, 1992), both of which may be confounding influences.

This paper forms an update of a previous study (West et al, 1993) and now includes 128 patients compared with the original 88 patients. In this report, for the first time, a multivariate analysis has been performed to study the independence of $SF_2$ as a prognostic factor for carcinoma of the cervix treated with radiotherapy.

*Received 12 February 1997*
*Revised 28 April 1997*
*Accepted 29 April 1997*

*Correspondence to:* C West

### MATERIALS AND METHODS

### Patient details

The patient details, treatment protocols and assay method have been described in detail elsewhere (West et al, 1993). The study was performed after South Manchester Medical Research Ethics Committee approval, and only women with stage I–III proven carcinoma of the cervix who gave informed consent were included in the study. Tumour histological type was mainly squamous cell carcinoma ($n$ = 118) with eight adenocarcinoma and two adeno-squamous cell carcinoma. The median patient age at the start of treatment was 51 years with a range of 23–78 years. Tumour size was measured either by magnetic resonance imaging (MRI) ($n$ = 39) to obtain an average diameter from three planes (anterior–posterior, lateral and craniocaudal) or by clinical examination ($n$ = 37). The median tumour diameter was 4 cm with a range of 1–8 cm. For tumours for which both MRI and clinical size data were available ($n$ = 26), the MRI figures were used. There was however no significant difference between the two measurements, and only 1 of the 26 tumours had a tumour that was greater than the median diameter by clinical examination but was smaller when measured by MRI. Treatment was with radical (i.e. with curative intent) radiotherapy alone, and follow-up times ranged from 2 to 5 years with a median of 47 months. Women suspected of pelvic recurrence within the radiation field were reassessed and the recurrence was confirmed histologically and/or using radiological techniques. The recurrences were divided into central (i.e. central pelvic recurrence), peripheral (i.e. those occurring at the edge of the radiation field) and metastatic. Recurrence on the pelvic side-wall was taken as being peripheral for external beam-irradiated tumours or as being metastatic disease for those treated solely with intracavitary irradiation.

### Clonogenic assay

Tumour specimens were received immediately before the commencement of radiotherapy. Samples were disaggregated using an enzyme cocktail containing 0.4 mg $ml^{-1}$ DNAase, 0.5 mg $ml^{-1}$ pronase and 0.5 mg $ml^{-1}$ collagenase for 1.5 h followed by 0.05% trypsin for 0.5 h. Single-cell suspensions were cultured using a soft agar clonogenic assay in Ham's F12 medium supplemented with 15% fetal calf serum, August rat red blood cells, 10 ng $ml^{-1}$ epidermal growth factor, 10 μg $ml^{-1}$ insulin, 0.5 μg $ml^{-1}$ hydrocortisone and 2.5 μg $ml^{-1}$ transferrin. Intrinsic radiosensitivity was determined as sensitivity to a single in vitro dose of 2 Gy radiation ($SF_2$) after irradiation with a $^{137}Cs$ γ-source with a dose rate of 3.8–4.2 Gy $min^{-1}$. $SF_2$ was calculated from the colony-forming efficiencies of control and irradiated samples after 4 weeks growth in an atmosphere of 5% carbon dioxide plus 5% oxygen plus 90% nitrogen. Larger samples were also irradiated with 3.5 Gy to obtain $SF_{3.5}$.

### Immunohistochemical analysis of tumour colonies

The characterization of cells growing in colonies was carried out using immunohistochemistry. Under a light microscope and using a glass pipette, colonies were plucked from agar. The colonies were exposed to trypsin and then the cells were placed onto slides using a cytocentrifuge. Cells were fixed at room temperature in acetone for 2 min. Cell staining was carried out with CAM5.3 (recognizing cytokeratins 8, 18 and 19; Becton-Dickenson) and CK1 (recognizing cytokeratins 6, 18; Dako) antibodies using the alkaline phosphatase–anti-alkaline phosphatase (APAAP) method. A mouse fibroblast strain (3T3) was used as a negative control.

### Statistical analysis

The Mann–Whitney *U*-test was used to test for the level of significance of differences between data sets. The probabilities of overall survival, locoregional control and metastasis-free survival were determined using univariate and bivariate (stratified) log-rank analysis, with the continuous variables grouped into two (above and below median values) or three (for disease stage and tumour grade) bands. As group boundaries corresponded to the medians ($SF_2$, age, volume), values falling on the boundaries meant that, in practice, the data subsets were not always exactly the same size. A stepwise multivariate Cox regression was also performed to test for the independence of $SF_2$ measurements from clinical parameters. A significance level of 0.05 was used throughout.

### RESULTS

Validation of the assay used in this study has been reported elsewhere (Davidson et al, 1990, 1992). Immunohistochemistry using low-molecular-weight cytokeratins was used to suggest the epithelial origin of colonies growing in agar. Nine tumours were examined using CAM5.2 (recognizing cytokeratins 8, 18 and 19) and CK1 (recognizing cytokeratins 6, 18) antibodies and all showed positive staining. Although the majority were positive using both stains, two tumours were CAM5.2 positive and CK1 negative, showing the importance of using multiple cytokeratin markers. Tumour colonies from two specimens were cultured in repeat experiments for transmission electron microscopy, and appearances were consistent with those of undifferentiated epithelial cells.

Over the period of this study, 241 tumour samples were received. Of these, 180 grew in culture representing an assay success rate of 75%, 53 failed to meet the criteria for successful growth (greater than ten colonies per control tube) and eight cultures became infected. For some tumours, there was only enough material for plating control tubes ($n$=13), and so the success rate for obtaining $SF_2$ values was 71%. Assay errors and success rates have also been described previously (West et al, 1989; Davidson et al, 1990; West et al, 1993). Only 128 of the 167 tumours were included in the study and the reasons for exclusion were: stage IV disease, palliative treatment, treatment with adjuvant chemotherapy. The mean, median and range of values for colony-forming efficiency (CFE) were: 0.20%, 0.06% and 0.004–2.90% respectively.

Table 1 summarizes the $SF_2$ values for the 128 patients included in the study. The median value for $SF_2$ was 0.42 with a range of 0.14–0.93. Eighty-five patients were alive at the time of analysis (including five intercurrent deaths at 11, 11, 18, 26 and 28 months), and these patients had a median $SF_2$ of 0.38, which was significantly lower than the value of 0.53 for 43 tumours from patients dead of disease and 0.54 for 30 patients who developed local recurrence. Five of the patients alive at the time of analysis had recurrent disease, and this was either local ($SF_2$ = 0.69, 0.79) or metastatic ($SF_2$ = 0.32, 0.39, 0.49). Excluding these patients from the 'alive' group did not affect the results of these analyses (median $SF_2$ value for 83 patients alive without recurrence = 0.38). There is one finding that differs from our earlier analyses (West et al, 1993). In the current analyses, the median tumour $SF_2$ value obtained for patients alive and well was not significantly different from the median for those with peripheral recurrence ($P$ = 0.21). In the previous analysis, tumours that recurred peripherally were significantly more radioresistant than the alive and well group.

Log-rank analysis of survival (Figure 1), stratifying according to the median value, showed that $SF_2$ was a significant prognostic factor for survival ($P$ = 0.0002), local control ($P$ = 0.0013) and metastasis-free survival ($P$ = 0.0039). This analysis was repeated with the $SF_2$ values divided into four quartiles. Patient survival decreased significantly with increasing tumour cell radioresistance

**Table 1** Summary of $SF_2$ values

| Patient group | $n$ | Median $SF_2$ | $P^a$ |
|---|---|---|---|
| All | 128 | 0.42 | |
| Alive | 85 | 0.38 | |
| Dead | 43 | 0.53 | <0.01 |
| Recurrence | 30 | 0.54 | 0.020 |
| Central recurrence | 15 | 0.57 | 0.020 |
| Peripheral recurrence | 18 | 0.47 | 0.21 |
| Metastases and recurrence | 38 | 0.52 | 0.010 |
| Metastases only | 21 | 0.49 | 0.070 |

[a]The level of significance, using the Mann–Whitney test, of the median $SF_2$ values for the various outcome groups vs the value for patients alive at the time of analysis.

© Cancer Research Campaign 1997

**Figure 1** Survival (**A**), local control (**B**) and metastasis-free survival (**C**) vs $SF_2$. The data from 128 patients have been stratified according to the median $SF_2$ value, and for all graphs the upper arm is for $SF_2$<0.42. The numbers of events per patients are given on each arm with 8 out of 32, 3 out of 31, 14 out of 30 and 17 out of 35 deaths in the four quartiles ($P = 0.0019$). This was accompanied with a significant increase in the number of patients with local recurrence (6 out of 32, 2 out of 31, 9 out of 30 and 13 out of 35 locoregional failures; $P = 0.005$) and decrease in metastasis-free survival (7 out of 32, 5 out of 31, 12 out of 30, 14 out of 35; $P = 0.02$). $SF_{3.5}$ was obtained for 67 patients. Using the median of this parameter to stratify patients, those with radioresistant tumours suffered more deaths and local recurrences than those with sensitive tumours, but the differences were not significant in this smaller dataset ($P = 0.06$ for survival, $P = 0.19$ local control, $P = 0.10$ for metastasis-free survival).

As expected, disease stage was a significant prognostic factor (Figure 2). Patient survival decreased with advancing stage with 9 out of 44, 18 out of 49 and 16 out of 35 deaths in stage I, II and III disease respectively ($P = 0.01$). Although not significant, local recurrence levels tended to increase with 7 out of 44, 12 out of 49 and 11 out of 35 locoregional failures for stage I, II and III disease respectively ($P = 0.11$). The number of patients developing metastases also increased with stage (8 out of 44, 18 out of 49, 12 out of 35; $P = 0.074$). Neither patient age ($n = 128$, $P = 0.85$), tumour grade ($n = 117$, $P = 0.52$) or tumour diameter ($n = 76$, $P = 0.35$) were significantly associated with overall survival. Also, age ($P = 0.17$, $P = 0.41$), grade ($P = 0.24$, $P = 0.83$) and diameter ($P = 0.21$, $P = 0.27$) were not prognostic for either recurrence or metastasis-free survival respectively.

In order to evaluate the independence of $SF_2$ as a prognostic factor, an evaluation was made of treatment outcome using bivariate log-rank analyses. Patients were stratified according to the median $SF_2$ value after allowing for stage (Figure 3), grade, age (Figure 4), colony-forming efficiency (CFE) and diameter (Figure 5). In all cases, $SF_2$ remained to be a significant prognostic variable (Table 2). From these analyses, it was found that tumour radiosensitivity was more important in stage III disease for younger patients and in larger tumours. A multivariate Cox regression analysis was also carried out. This indicated that only $SF_2$ and stage were significant independent prognostic factors in this data set (Table 3).

### DISCUSSION

Two recent studies have reported the growth of fibroblasts in soft agar clonogenic assays of tumours (Lawton et al, 1994; Stausbol et al, 1995). In a study of three human lung tumours, the majority (67–100%) of colonies examined after culture stained positively with a fibroblast marker (Lawton et al, 1994). For the same samples, only 34–75% of colonies stained positively with cytokeratin antibodies. The latter observation suggests cross-reactivity of antibody staining, e.g. for one tumour eight out of eight and three out of four colonies were positive for a fibroblast *and* a cytokeratin marker respectively. In our own work on cervix tumours, all the colonies from nine tumours examined showed positive low-molecular-weight cytokeratin staining, which is consistent with an epithelial origin. However, it is recognized that cytokeratins have been shown to cross-react with non-epithelial tissues, e.g. myoepithelial. It is also of note that we found that a negative result with one source of cytokeratin antibodies could be reversed by staining with antibodies from another source, indicating the necessity of using multiple low-molecular-weight cytokeratin markers to identify cell types with a reasonable degree of certainty.

Indirect evidence for the tumour origin of colonies comes from the demonstration that CFE was a prognostic factor for recurrence-free survival in cervix patients. CFE is a very variable parameter, and we have shown that measurements are associated with significant intratumour and interexperiment variation (Davidson et al, 1992). Despite this variation, CFE was shown to be a significant prognostic factor for local control in this analysis and in an earlier analysis on fewer patients (West et al, 1993). In addition, within the multivariate analysis, after allowing for tumour $SF_2$ and stage, CFE showed borderline significance for local control (Table 3). CFE was not prognostic for overall survival. These findings are consistent with CFE reflecting tumour stem cell content, which would be more likely to predict for local control than for overall survival in radiotherapy-treated disease (a CFE reflecting fibroblast growth (see above) would not be expected to relate to treatment outcomes).

© Cancer Research Campaign 1997

**Figure 2** Survival (**A**) and local control (**B**) vs disease stage. Patients were divided into stage I, II and III disease with 44, 49 and 35 patients, respectively, in each group

**Figure 3** Survival (**A–C**) and local control (**D–F**) vs $SF_2$, stratified according to the median; for all graphs the upper arm is for $SF_2$ <0.42. Patients were divided into stage I, II and III disease with 44, 49 and 35 patients, respectively, in each group

This updated analysis confirms the findings from earlier analyses performed on 51 (West et al, 1991) and 88 (West et al, 1993) patients. Considering the current interest in radiosensitivity testing, there is a dearth of clinical studies measuring tumour intrinsic radiosensitivity. However, as summarized in the Introduction, there are several small studies that do confirm our observation that radiosensitive tumours are more responsive to radiotherapy (reviewed in West 1994, 1995). The importance of tumour radiosensitivity was highlighted originally by Fertil and Malaise (1981). In another paper (Malaise et al, 1987), these authors published an analysis of the capacity of different survival levels to identify significant differences between tumour radio-responsiveness classes. The relationship was shown to be dose dependent over the range 1–6 Gy with a bell-shaped curve and a maximum at 1.5 Gy. The finding reported here for primary human tumours that $SF_{3.5}$ was less effective than $SF_2$ as a prognostic factor for treatment outcomes supports the observations made on tumour cell lines.

Obviously, it is important to examine the independence of any putative prognostic factor. This has been highlighted in our own work in a study of pretreatment measurements of serum markers (CA125, SCC, TPA) in carcinomas of the cervix. Although all

© Cancer Research Campaign 1997

**Figure 4** Survival (**A** and **B**) and local control (**C** and **D**) vs $SF_2$, stratified according to the median (upper arms $SF_2$<0.42). Patients were divided into those above (**B** and **D**) and below (**A** and **C**) median age of 51. Data from 128 patients

**Figure 5** Survival (**A** and **B**) and local control (**C** and **D**) vs $SF_2$, stratified according to the median (upper arms are for $SF_2$, <0.42). Patients were divided into those above (**B** and **D**) and below (**A** and **C**) the median tumour diameter of 4 cm. Data from 76 patients

© Cancer Research Campaign 1997

**Table 2** The independence of tumour $SF_2$ as a prognostic factor in carcinoma of the cervix treated with radiotherapy

| | *n* | Survival[a] | Local control[a] | Metastasis-free survival[a] |
|---|---|---|---|---|
| Stage | 128 | 0.0002 | 0.0014 | 0.0026 |
| Grade | 117 | 0.0005 | 0.0037 | 0.0051 |
| Age | 128 | 0.0002 | 0.0007 | 0.0021 |
| CFE | 128 | 0.0002 | 0.0016 | 0.0023 |
| Diameter | 76 | 0.0098 | 0.0006 | 0.028 |

[a]The level of significance of $SF_2$ as a prognostic factor after allowing for the listed parameters. Stratified log-rank analysis was used.

**Table 3** Cox multivariate analysis showing the relative risk (RR) for survival and local recurrence-free survival

| Variable | Survival | | Local control | |
|---|---|---|---|---|
| | RR | *P* | RR | *P* |
| $SF_2$ ($n$ = 128) | 3.4 | <0.001 | 6.4 | <0.001 |
| Stage | | | | |
| II vs I | 2.1 | 0.007 | NS | 0.28 |
| III vs I | 3.6 | | | |
| CFE | NS | 0.93 | NS | 0.09 |
| $SF_2$ ($n$ = 68) | 4.0 | 0.001 | 16.2 | <0.001 |
| Stage | | | | |
| II vs I | 1.6 | 0.051 | 0.72 | 0.069 |
| III vs I | 4.1 | | 4.5 | |

Upper values considered $SF_2$, stage, CFE and age as candidates for the Cox model while the lower values considered $SF_2$, stage, CFE, age, grade and diameter, but with a smaller dataset. Only parameters that had a significant prognostic value entered the Cox model. Non-significant terms (NS) had an RR not significantly different from 1.

three markers were prognostic for treatment outcomes, after allowing for disease stage in multivariate analyses, prognostic significance was predominantly lost (Sproston et al, 1995). In the current study with 128 women, the higher patient numbers have now permitted multivariate analyses to be performed.

From the analyses, several observations can be made that add strength to the finding that tumour radiosensitivity is an important determinant of outcome after radiotherapy. The data show that tumour radiosensitivity is more important in stage III disease and for bulky tumours. The finding is consistent with a greater stem cell content of larger tumours, resulting in a greater risk of loss of tumour control perhaps because of insufficient radiation dose, probably particularly so at the edge of radiation fields (Figure 5B). For small-volume disease, tumour radiosensitivity would be less important because of fewer tumour cells on the edge of radiotherapy fields (Figure 5A). The same explanation can be used in describing the importance of tumour radiosensitivity for different stages of disease. The finding that tumour radiosensitivity is more important for younger women is interesting and perhaps suggests some hormonal influence for differences in pre- and postmenopausal women.

In this study, neither tumour size, patient age nor histological grade were significant prognostic factors. A number of studies have shown that tumour size is an important prognostic variable in carcinoma of the cervix treated with radiotherapy (e.g. Fyles et al, 1995). Possible reasons for the lack of significance of tumour size in our study are the inclusion of all three stages in the analysis (i.e. different treatment methods), stratifying data according to the median value and the low numbers of evaluable cases. Although there are some reports of patient age and tumour grade as being significant prognostic factors in cervix tumours (e.g. Prempree et al, 1983), these have not been consistent findings (e.g. Russell et al, 1989).

Our work shows that tumour radiosensitivity is important, but it does not address the issue of whether tumour radiosensitivity testing will be of clinical value. The clonogenic assay used in this study is probably too laborious to be of routine clinical use. In addition, it takes 4 weeks to generate results with only a 70% success rate. There is clearly a need for a rapid and reliable test. The future of tumour radiosensitivity testing will also be dependent on the clinician's ability to offer alternative treatment. In theory, dose escalation to radioresistant tumours could lead to impressive increases in local control (West and Hendry, 1992), but this strategy will be dependent on having a highly sensitive and specific assay for normal tissue radiosensitivity. For some tumour sites, e.g. head and neck cancers, extensive surgery might be an alternative treatment to radiotherapy for some radioresistant tumours. Radiosensitivity testing might also be used to examine novel chemotherapeutic agents, and the potential of this is illustrated in Figure 6 in which the importance of disease stage is highlighted in patients with radioresistant tumours.

In conclusion, in this work we have confirmed our own earlier observations that tumour radiosensitivity is an important determinant of outcome after radiotherapy. In addition, the study has shown that tumour radiosensitivity is independent of other established and putative prognostic variables.

© Cancer Research Campaign 1997

**Figure 6** Survival vs disease stage for patients with radiosensitive (**A**) or radioresistant (**B**) tumours. Patients were divided into stage I, II and III disease with 44, 49 and 35 patients, respectively, in each group

### ACKNOWLEDGEMENTS

The authors thank Mrs Paula Berry and Mrs Deepti Wilks for technical assistance and Professor Jolyon Hendry for useful discussions.

### REFERENCES

Allalunis-Turner MJ, Day RS, Pearcey RG and Urtasum RC (1992) Radiosensitivity testing in gynecological tumors and malignant gliomas. In *Radiation Research: A Twentieth Century Perspective*, Dewey WC, Edington M, Fry RJM, Hall EJ and Whitmore GF. (eds), pp. 712–715. Academic Press: San Diego

Brock WA, Brown BW, Goefpert H and Peters LJ (1992) *In vitro* radiosensitivity of tumor cells and local tumor control by radiotherapy. In *Radiation Research: A Twentieth Century Perspective*, Dewey WC, Edington M, Fry RJM, Hall EJ and Whitmore GF. (eds), pp. 696–699. Academic Press: San Diego

Davidson SE, West CML, Roberts SA, Hendry JH and Hunter RD (1990) Radiosensitivity testing of primary cervical carcinoma: evaluation of intra- and inter-tumour heterogeneity. *Radiother Oncol* **18**: 349–356

Davidson SE, West CML and Hunter RD (1992) Lack of association between *in vitro* clonogenic growth of human cervical carcinoma and tumour stage, differentiation, patient age, host cell infiltration of patient survival. *Int J Cancer* **50**: 10–14

Fertil B and Malaise EP (1981) Inherent cellular radiosensitivity as a basic concept for human tumor radiotherapy. *Int J Radiat Oncol Biol Phys* **7**: 621–629

Fyles AW, Pintilie M, Kerkbride P, Levin W, Manchul LA and Rawlings GA (1995) Prognostic factors in patients with cervix cancer treated by radiation therapy: results of a multiple regression analysis. *Radiother Oncol* **35**: 107–117

Girinsky T, Bernhai A, Lubin R, Tavakoli-Razavit T, Baker F, Janot F, Wibault P, Cosset J-M, Duvillard P, Duverger A and Fertil B (1994) *In vitro* parameters and treatment outcome in head and neck cancers treated with surgery and/or radiation: cell characterization and correlations with local control and overall survival. *Int J Radiat Oncol Biol Phys* **30**: 789–794

Hinkley HJ and Bosanquet AG (1992) The *in vitro* radiosensitivity of lymphocytes from chronic lymphocytic leukaemia using the differential staining cytotoxicity (DiSC) assay. II – Results on 40 patients. *Int J Radiat Biol* **61**: 111–121

Lawton PA, Hodgkiss RJ, Eyden BP and Joiner MC (1994) Growth of fibroblasts as a potential confounding factor in soft agar clonogenic assays for tumor cell radiosensitivity. *Radiother Oncol* **12**: 218–225

Malaise EP, Fertil B, Deschavanne PJ, Chavaudra N and Brock WA (1987) Initial slope of radiation survival curves is characteristic of the origin of primary and established cultures of human tumor cells and fibroblasts. *Radiat Res* **111**: 319–333

Prempree T, Patanaphan V, Sewchand W and Scott RM (1983) The influence of patients' age and tumor grade on the prognosis of carcinoma of the cervix. *Cancer* **51**: 1764–1771

Ramsay J, Ward R and Bleehen NM (1992) Radiosensitivity testing of human malignant gliomas. *Int J Radiat Oncol Biol Phys* **24**: 675–680

Russell JM, Blair V and Hunter RD (1987) Cervical carcinoma: prognosis in younger patients. *Br Med Journal* **295**: 300–303

Schwartz JL, Beckett MA, Mustafir R, Vaughan ATM and Weichselbaum RR (1992) Evaluation of different in vitro assays of inherent sensitivity as predictors of radiotherapy response. In *Radiation Research: A Twentieth Century Perspective*, Dewey WC, Edington M, Fry RJM, Hall EJ and Whitmore GF. (eds), pp. 716–721. Academic Press: San Diego

Shibamoto Y, Shibato T, Miyatake S, Oda Y, Manabe T, Ohshio G, Yagi K, Streffer C, Takahashi M and Abe M (1994) Assessment of the proliferative activity and radiosensitivity of human tumours using the cytokinesis-block micronucleus assay. *Br J Cancer* **70**: 67–71

Sproston ARM, Roberts SA, Davidson SE, Hunter RD and West CML (1995) Serum tumour markers in carcinoma of the uterine cervix and outcome following radiotherapy. *Br J Cancer* **72**: 1536–1540

Stausbol B, Nielsen OS, Bentzen SM and Overgaard J (1995) Selective assessment of in vitro radiosensitivity of tumour cells and fibroblasts from single tumour biopsies using immunocytochemical identification of colonies in the soft agar clonogenic assay. *Radiother Oncol* **37**: 87–99

Taghian A, Ramsay J, Allalunis-Turner J, Budach W, Gioioso D, Pardo F, Okunieff P, Bleehen N, Urtasun R and Suit H (1993) Intrinsic radiation sensitivity may not be the major determinant of the poor clinical outcome of glioblastoma multiforme. *Int J Radiat Oncol Biol Phys* **25**: 253–249

Vaughan ATM, Anderson P, Wallace DMA, Beaney RP and Lunch TH (1993) Local control of T2/3 transitional cell carcinoma of bladder is correlated to differences in DNA supercoiling: evidence for two discrete tumor populations. *Cancer Res* **53**: 2300–2303

West CML (1994) Predictive assays in radiation therapy. *Adv Radiat Biol* **18**: 149–180

West CML (1995) Intrinsic radiosensitivity as a predictor of patient response to radiotherapy. *Br J Radiol* **68**: 827–837

West CML and Hendry JH (1992) Intrinsic radiosensitivity as a predictor of patient response to radiotherapy. *Br J Radiol* **Suppl 24**: 146–152

West CML, Davidson SE, Hunter RD, James RD, Schofield PF, Moore JV and Hendry JH (1989) Surviving fraction at 2 Gy for colony-forming cells in primary cultures of human cervical and colorectal tumours. *Am Assoc Phys Med* **7**: 86–95

West CML, Davidson SE, Hendry JH and Hunter RD (1991) Prediction of cervical carcinoma resonse to radiotherapy. *Lancet* **338**: 818

West CML, Davidson SE, Roberts SA and Hunter RD (1993) Intrinsic radiosensitivity and prediction of patient response to radiotherapy for carcinoma of the cervix. *Br J Cancer* **68**: 819–823

*© Cancer Research Campaign 1997*


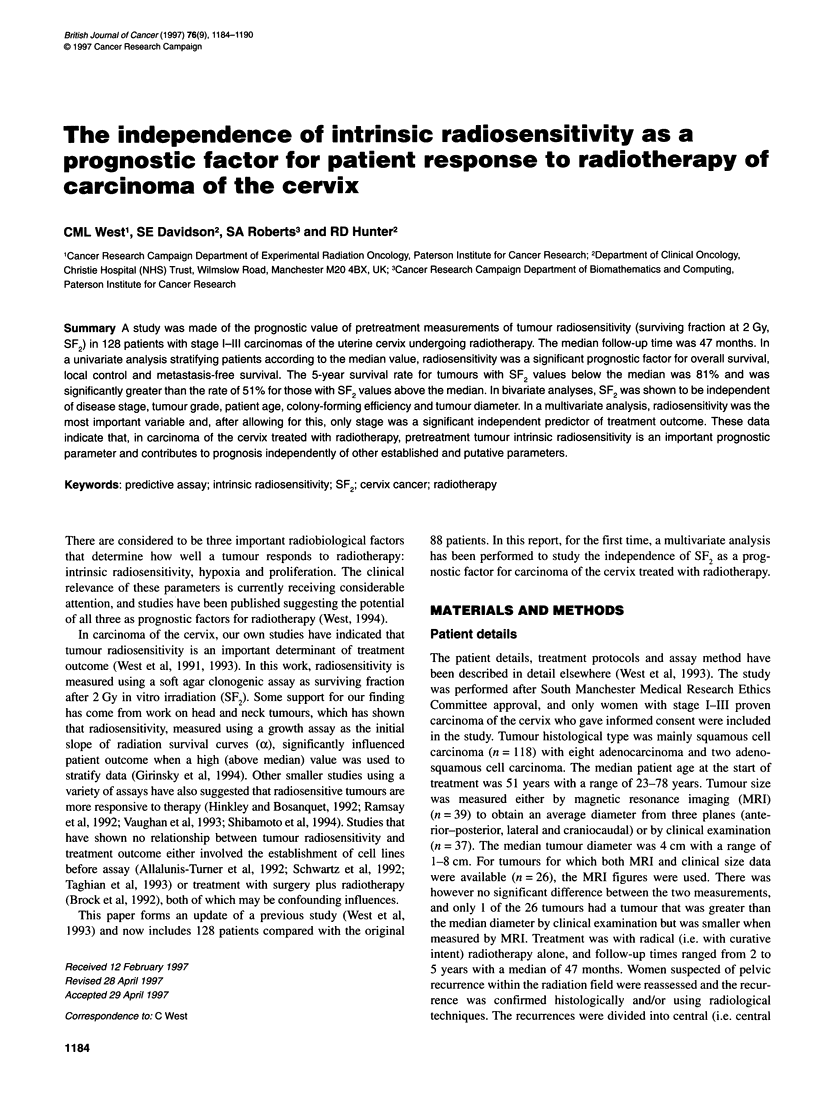

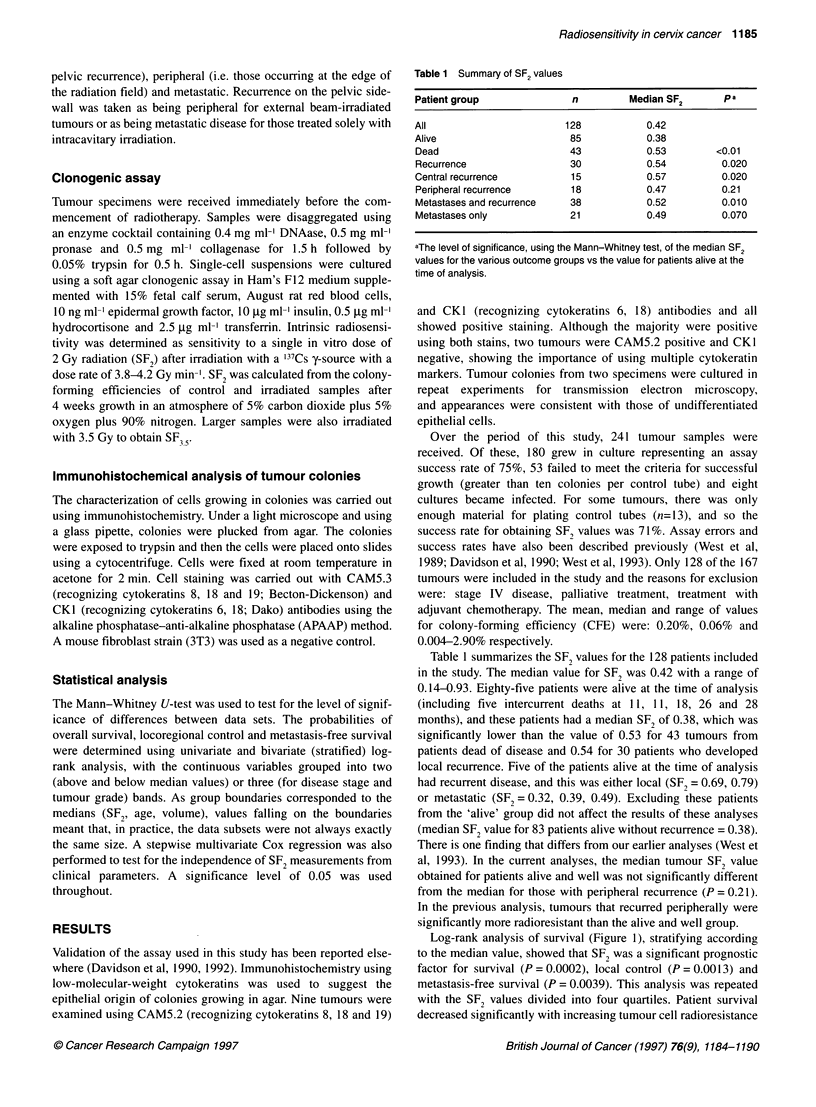

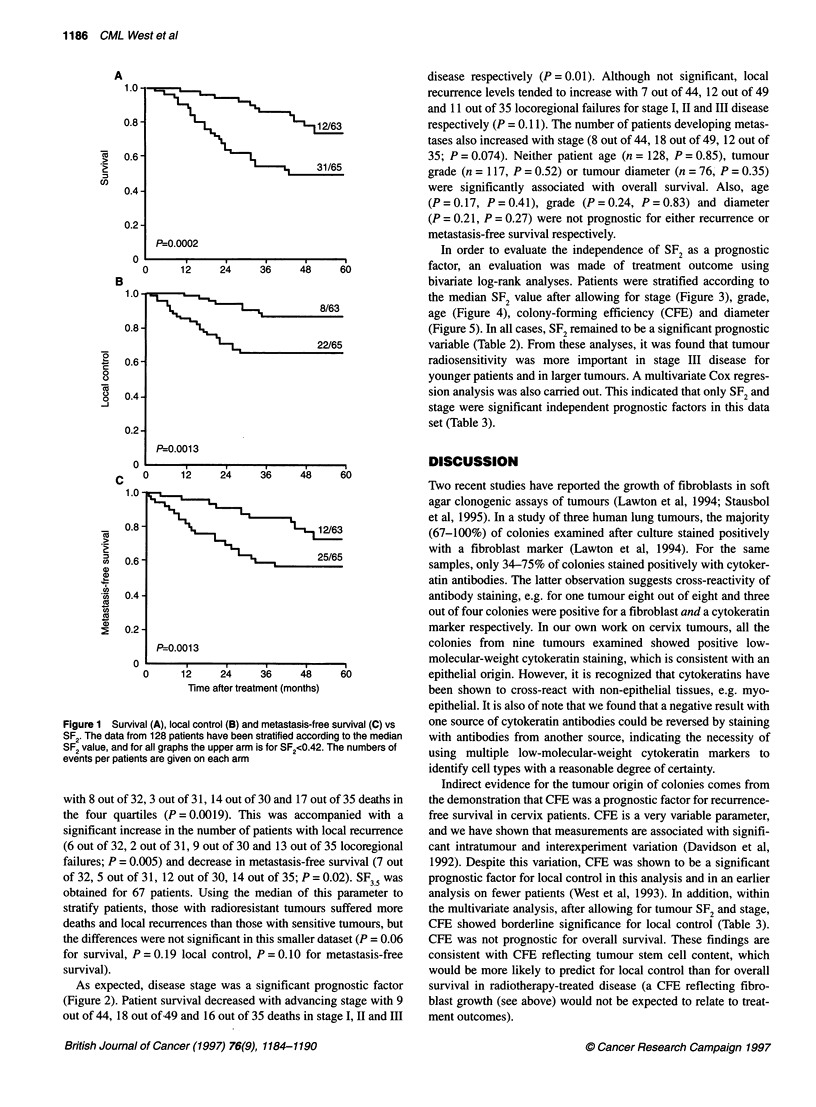

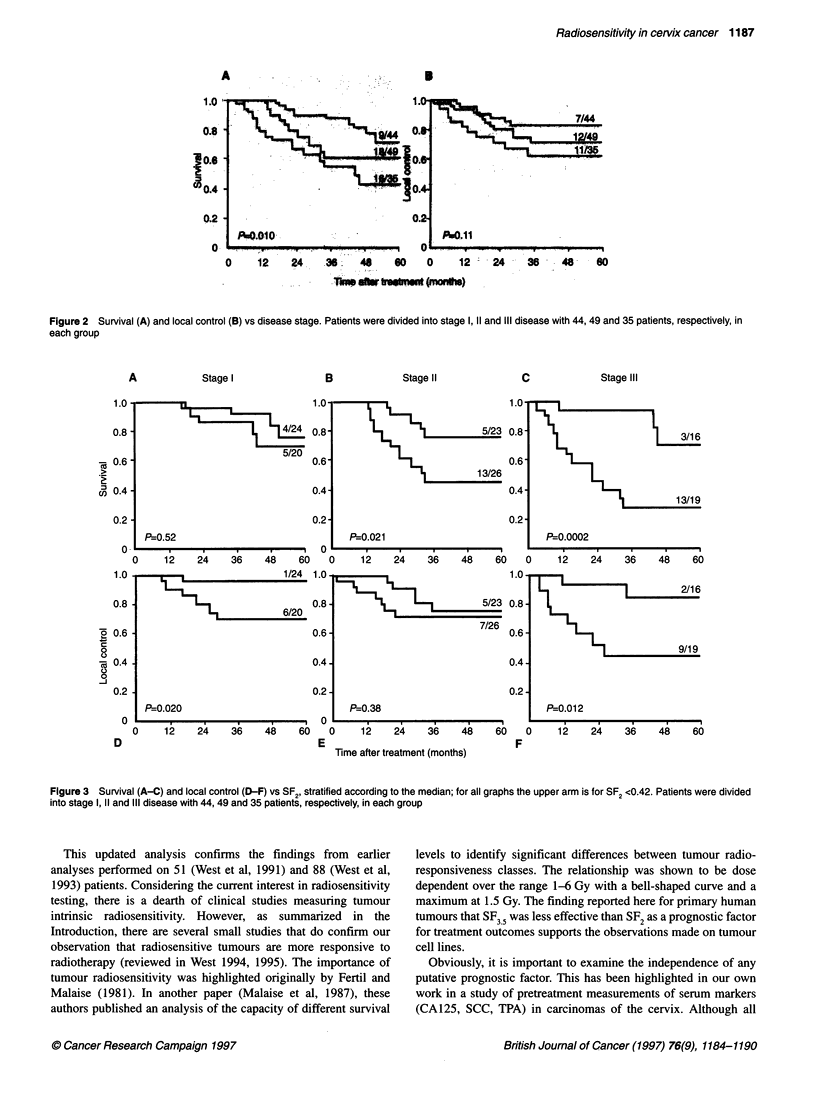

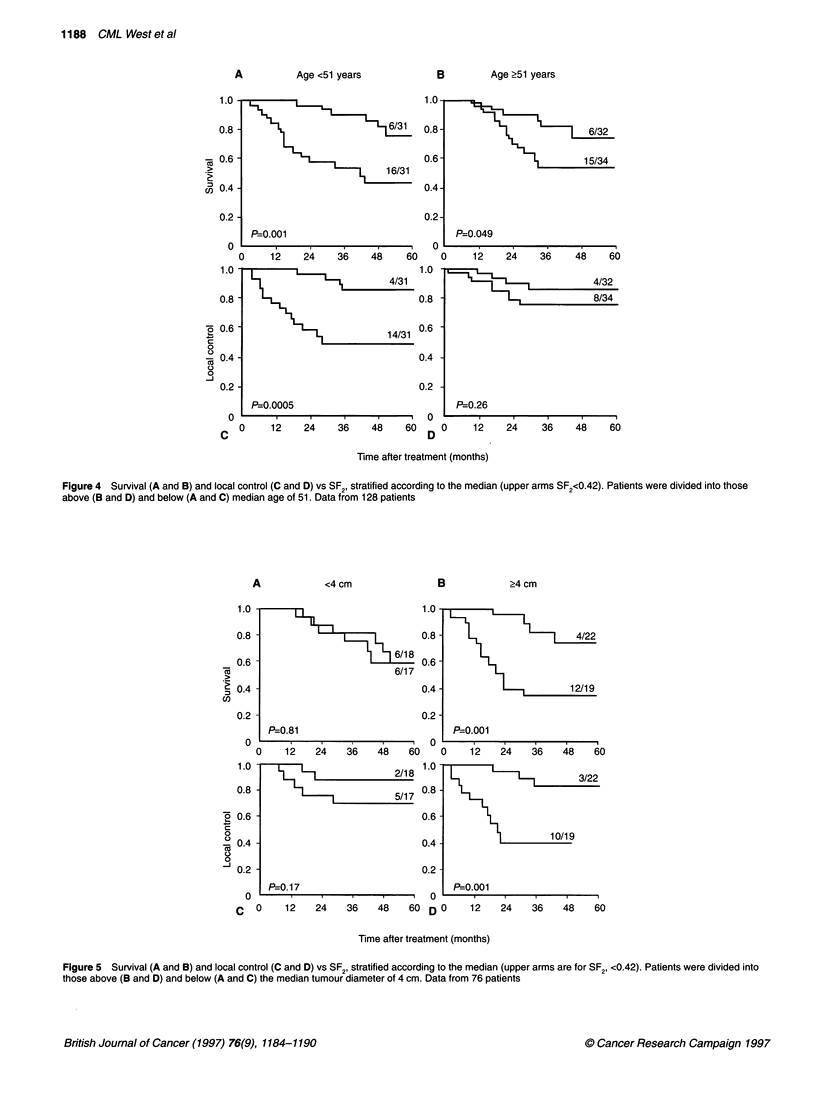

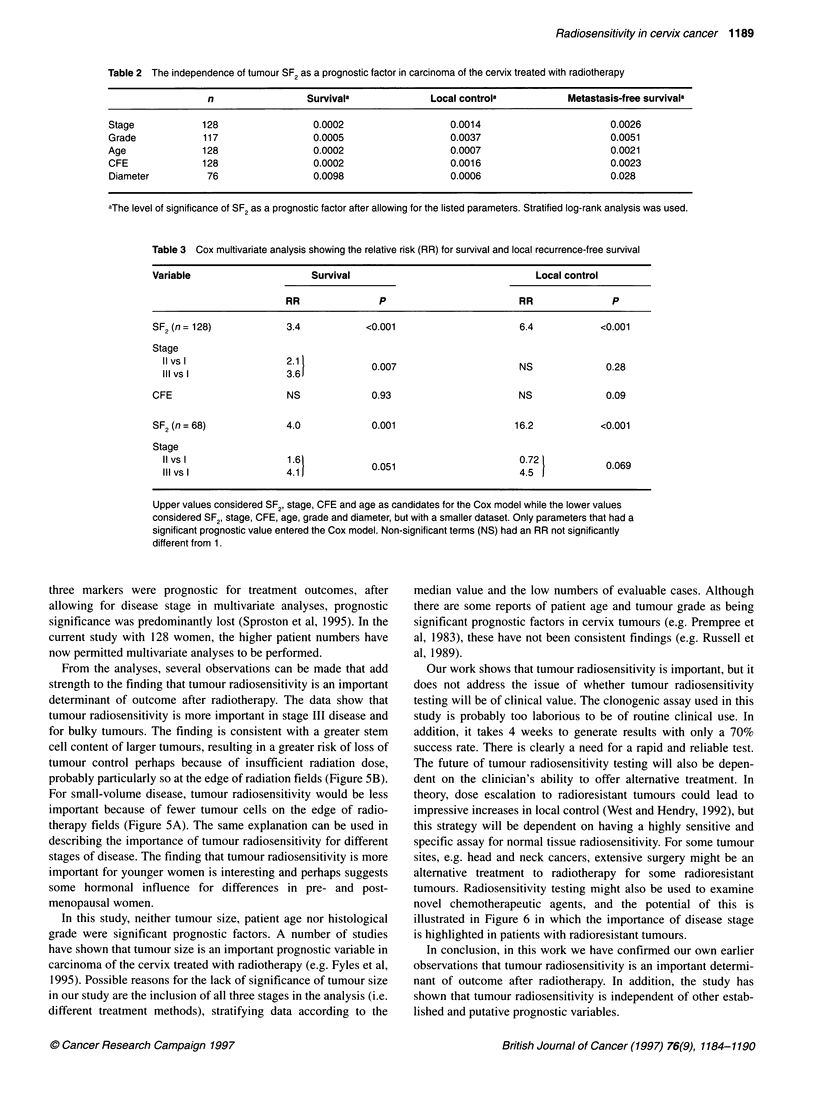

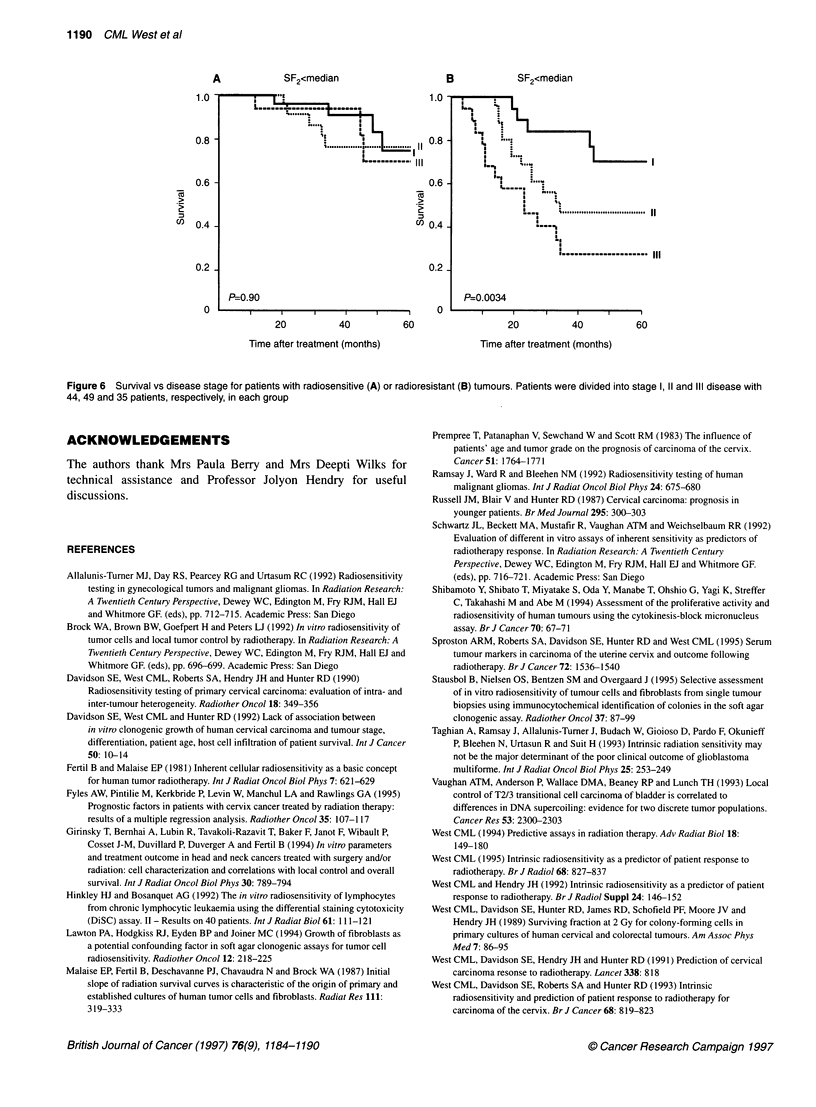


## References

[OCR_00830] Davidson S. E., West C. M., Hunter R. D. (1992). Lack of association between in vitro clonogenic growth of human cervical carcinoma and tumour stage, differentiation, patient age, host cell infiltration or patient survival.. Int J Cancer.

[OCR_00825] Davidson S. E., West C. M., Roberts S. A., Hendry J. H., Hunter R. D. (1990). Radiosensitivity testing of primary cervical carcinoma: evaluation of intra- and inter-tumour heterogeneity.. Radiother Oncol.

[OCR_00837] Fertil B., Malaise E. P. (1981). Inherent cellular radiosensitivity as a basic concept for human tumor radiotherapy.. Int J Radiat Oncol Biol Phys.

[OCR_00841] Fyles A. W., Pintilie M., Kirkbride P., Levin W., Manchul L. A., Rawlings G. A. (1995). Prognostic factors in patients with cervix cancer treated by radiation therapy: results of a multiple regression analysis.. Radiother Oncol.

[OCR_00846] Girinsky T., Bernheim A., Lubin R., Tavakoli-Razavi T., Baker F., Janot F., Wibault P., Cosset J. M., Duvillard P., Duverger A. (1994). In vitro parameters and treatment outcome in head and neck cancers treated with surgery and/or radiation: cell characterization and correlations with local control and overall survival.. Int J Radiat Oncol Biol Phys.

[OCR_00853] Hinkley H. J., Bosanquet A. G. (1992). The in vitro radiosensitivity of lymphocytes from chronic lymphocytic leukaemia using the differential staining cytotoxicity (DiSC) assay. II--Results on 40 patients.. Int J Radiat Biol.

[OCR_00858] Lawton P. A., Hodgkiss R. J., Eyden B. P., Joiner M. C. (1994). Growth of fibroblasts as a potential confounding factor in soft agar clonogenic assays for tumour cell radiosensitivity.. Radiother Oncol.

[OCR_00863] Malaise E. P., Fertil B., Deschavanne P. J., Chavaudra N., Brock W. A. (1987). Initial slope of radiation survival curves is characteristic of the origin of primary and established cultures of human tumor cells and fibroblasts.. Radiat Res.

[OCR_00869] Prempree T., Patanaphan V., Sewchand W., Scott R. M. (1983). The influence of patients' age and tumor grade on the prognosis of carcinoma of the cervix.. Cancer.

[OCR_00874] Ramsay J., Ward R., Bleehen N. M. (1992). Radiosensitivity testing of human malignant gliomas.. Int J Radiat Oncol Biol Phys.

[OCR_00878] Russell J. M., Blair V., Hunter R. D. (1987). Cervical carcinoma: prognosis in younger patients.. Br Med J (Clin Res Ed).

[OCR_00890] Shibamoto Y., Shibata T., Miyatake S., Oda Y., Manabe T., Ohshio G., Yagi K., Streffer C., Takahashi M., Abe M. (1994). Assessment of the proliferative activity and radiosensitivity of human tumours using the cytokinesis-block micronucleus assay.. Br J Cancer.

[OCR_00896] Sproston A. R., Roberts S. A., Davidson S. E., Hunter R. D., West C. M. (1995). Serum tumour markers in carcinoma of the uterine cervix and outcome following radiotherapy.. Br J Cancer.

[OCR_00901] Stausbøl-Grøn B., Nielsen O. S., Møller Bentzen S., Overgaard J. (1995). Selective assessment of in vitro radiosensitivity of tumour cells and fibroblasts from single tumour biopsies using immunocytochemical identification of colonies in the soft agar clonogenic assay.. Radiother Oncol.

[OCR_00907] Taghian A., Ramsay J., Allalunis-Turner J., Budach W., Gioioso D., Pardo F., Okunieff P., Bleehen N., Urtasun R., Suit H. (1993). Intrinsic radiation sensitivity may not be the major determinant of the poor clinical outcome of glioblastoma multiforme.. Int J Radiat Oncol Biol Phys.

[OCR_00913] Vaughan A. T., Anderson P., Wallace D. M., Beaney R. P., Lynch T. H. (1993). Local control of T2/3 transitional cell carcinoma of bladder is correlated to differences in DNA supercoiling: evidence for two discrete tumor populations.. Cancer Res.

[OCR_00939] West C. M., Davidson S. E., Hendry J. H., Hunter R. D. (1991). Prediction of cervical carcinoma response to radiotherapy.. Lancet.

[OCR_00943] West C. M., Davidson S. E., Roberts S. A., Hunter R. D. (1993). Intrinsic radiosensitivity and prediction of patient response to radiotherapy for carcinoma of the cervix.. Br J Cancer.

[OCR_00928] West C. M., Hendry J. H. (1992). Intrinsic radiosensitivity as a predictor of patient response to radiotherapy.. BJR Suppl.

[OCR_00924] West C. M. (1995). Invited review: intrinsic radiosensitivity as a predictor of patient response to radiotherapy.. Br J Radiol.

